# The Genome-Scale Integrated Networks in Microorganisms

**DOI:** 10.3389/fmicb.2018.00296

**Published:** 2018-02-23

**Authors:** Tong Hao, Dan Wu, Lingxuan Zhao, Qian Wang, Edwin Wang, Jinsheng Sun

**Affiliations:** ^1^Tianjin Key Laboratory of Animal and Plant Resistance, College of Life Sciences, Tianjin Normal University, Tianjin, China; ^2^Cumming School of Medicine, University of Calgary, Calgary, AB, Canada; ^3^Tianjin Bohai Fisheries Research Institute, Tianjin, China

**Keywords:** integrated network, metabolic network, regulatory network, signal transduction network, microorganism

## Abstract

The genome-scale cellular network has become a necessary tool in the systematic analysis of microbes. In a cell, there are several layers (i.e., types) of the molecular networks, for example, genome-scale metabolic network (GMN), transcriptional regulatory network (TRN), and signal transduction network (STN). It has been realized that the limitation and inaccuracy of the prediction exist just using only a single-layer network. Therefore, the integrated network constructed based on the networks of the three types attracts more interests. The function of a biological process in living cells is usually performed by the interaction of biological components. Therefore, it is necessary to integrate and analyze all the related components at the systems level for the comprehensively and correctly realizing the physiological function in living organisms. In this review, we discussed three representative genome-scale cellular networks: GMN, TRN, and STN, representing different levels (i.e., metabolism, gene regulation, and cellular signaling) of a cell’s activities. Furthermore, we discussed the integration of the networks of the three types. With more understanding on the complexity of microbial cells, the development of integrated network has become an inevitable trend in analyzing genome-scale cellular networks of microorganisms.

## Introduction

With the development of bioinformatics and system biology, large-scale cellular network comes into the sight of researchers. Bioinformatics, based on data processing, model construction and theoretical analysis, integrates information from different molecular levels to understand how the biological system works. According to the types of biological information processing encoded in the network, the cellular networks have been classified into different types: genome-scale metabolic network (GMN), transcriptional regulatory network (TRN), and signal transduction network (STN). The most well-studied large-scale biological network is GMN, which is a fundamental framework in systems metabolic engineering (Kim et al., 2015). With the first GMN constructed for *Haemophilus influenzae* Rd (Edwards and Palsson, 1999), the current GMN allows systematic level predictions of metabolism in a variety of organisms (Yilmaz and Walhout, 2017). The main concept of transcriptional control was established in bacterial system by Jacob and Monod (1961). In the past decades, the development of genomic technology and computational biology promotes the construction of large-scale TRNs (Brent, 2016). The TRN is composed of the interactions between different transcriptional factors (TFs) and target genes. A TF, which is encoded by a gene itself, may influence the expression of one or more target genes, which may subsequently give rise to the expression change of a serial of proteins or genes. The STN is different from the TRN in network structures and timescales. The STN contains protein–protein and protein–gene interactions, which includes multiple routes of rapid cell response to the external stimuli, whereas the TRN may need to produce sustained patterns of cellular activity over time (Babu et al., 2004; Papin et al., 2005). On the other hand, some proteins in the STN are TFs, which indicates some genes/proteins are in common between STNs and TRNs. The detailed comparisons of these networks have been described in the review (Wang et al., 2007).

From a system point of view, different kinds of biological networks are not working alone, but cooperate with each other to undertake their functions. Integrated network studies will build a more realistic model by investigating the interacting relationships and interacting effects among organism’s different information processing components in its system. This kind of models has an important sense to the theoretical research of living systems and the construction of genetic engineering strains (Wang et al., 2010). In this article, we discussed the research progress about the integrated networks in microorganisms.

## Cellular Network

Cellular network analysis has become a hot research area in bioinformatics and system biology; it utilizes computer model and experimental data to analyze complex biological system in a global view, and offers guidance and expectation for *in vivo* experiments (Wu and Ma, 2014). Due to the complexity of the biological system, researchers have classified cellular networks into GMN, TRN, and STN based on the types of information processing of biological molecules.

### Genome-Scale Metabolic Network

Due to the advances of genome sequencing, high-throughput data have been rapidly produced, which drives a transition from the traditional biology research. On the basis of genome sequencing and annotations in huge amounts of data, metabolic network reconstruction in a genome-scale has been developed rapidly (Francke et al., 2005; Notebaart et al., 2006). Currently, GMN has become an indispensable tool for studying the biological metabolic system (Pal et al., 2006; Feist and Palsson, 2008). It has important applications on designing classic paths of metabolic engineering, inverse metabolites synthesis, metabolic flux analysis, evolution analysis of metabolic pathways between different species, mining omic data, and identifying of the marks in enzyme engineering (Soh and Hatzimanikatis, 2010). GMN construction is based on genomic sequences, combining with genes, enzyme reactions, metabolic databases and related experimental data, to quantitatively study the metabolic processes of living organisms from a systematic perspective. All biochemical reactions in the cell have been included as a network and the GMN reflects the interactions between all the compounds involved in the metabolic processes and all the catalytic enzymes. The construction of a GMN allows an in-depth functional analysis of the biological metabolic system, which is different from the traditional approach analysis or biological response analysis, but try to understand the whole metabolic system from the systematic view. GMN brings a more comprehensive and accurate insight into cell metabolism of the whole system and the interaction relationships between different metabolic processes. On the other side, the topology of the metabolic networks among many organisms can reflect the dynamics of the metabolic system evolution, which can help us understand the history of life evolution in the context of metabolism (Ravasz et al., 2002; Stelling et al., 2002; Zhao, 2008; Deyasi et al., 2015). In all the genome-scale biological networks, GMN is the most extensive and deepest studied network, with its construction procedures generally normalized in Palsson’s review (Thiele and Palsson, 2010). The process of constructing of a metabolic network mainly consists of four parts, including data collection, relationship model establishment, data curation, and transformation into a mathematical model (Thiele and Palsson, 2010). To date, the construction of metabolic network has been able to realize some degree of automation, and therefore, 100s of metabolic networks in different organisms have been constructed (Hao et al., 2012).

Genome-scale metabolic network can be used to simulate the growth of organisms. Among the GMNs, the most accurate, comprehensive and classical model in microorganisms is the GMN of *Escherichia coli* named *i*JO1366, which was constructed by Palsson’s group in 2011. The model achieved 67.7 and 96% accuracies for the prediction of essential and non-essential genes in *E. coli*. It is capable of simulating the growth of *E. coli* on 334 kinds of nutrients (Orth et al., 2011). Recently, a novel updated GMN of *Clostridium difficile* which called *i*CDF834 has been presented. This network was constructed based on the model *i*MLTC806cdf and transcriptome data, which detailed the gene expression of the bacteria in various environments. It is worth mentioning that the synonymous codon usage bias was introduced into the model to remedy the inconsistence between gene expression and protein abundance, which is the first time that codon has been integrated into a GMN. The model achieved a quite high (92.3%) accuracy in predicting gene essentiality (Kashaf et al., 2017).

The GMN can be used to guide the metabolic engineering experiments. Using *Bacillus subtilis* as an example, Hao et al. (2013) constructed a GMN of *B. subtilis*, named *i*BSU1147. The model has been used to successfully predict the yields of four industrial products produced by *B. subtilis* [i.e., riboflavin, (R,R)-2,3-butanediol, cellulase Egl-237, and isobutanol]. The results have provided important guidance for the *in vivo* experiments (Hao et al., 2013). Recently, Piubeli et al. (2018) constructed a GMN *i*FP764 of halophilic bacterium *Chromohalobacter salexigens* to explore the cell factory for producing ectoine. This model was constructed based on the experimental data, genome sequences and re-annotation of metabolic genes. The GMN is capable of simulating the metabolic situation of *C. salexigens* in low and high yield of ectoines. The salinity-specific essential genes and the patterns of correlated reactions in central carbon and nitrogen metabolisms response to the change of salinity were also simulated. The network is a useful tool to improve the production of ectoines with bacteria (Piubeli et al., 2018).

The GMN also has an important value for drug discovery. Chen et al. (2015) constructed a GMN of *Treponema pallidum. T. pallidum* has a very specific metabolic network compared to those of other bacterial pathogens. It lacks the oxidative phosphorylation tricarboxylic and acid cycle pathways as well as is incapable of synthesizing enzyme cofactors, fatty acids, and most amino acids. By analyzing topological structure and minimal cut sets of the network, they found that some hub reactions in pyrimidine and purine metabolisms play significant roles in *T. pallidum*, which may be helpful drug targets in the treatment of syphilis, a sexually transmitted infection caused by the *T. pallidum* (Chen et al., 2015). In the same year, Steinway et al. (2015) constructed a GMN of intestinal bacteria based on experimental data. This network summarized the relationships between clindamycin and clostridium infection. Based on the analysis of topological and chemical properties of the network, the drug targets could be screened using the GMN, which can be used in the design of the drug-molecule model (Cong, 2010) and subsequently be applied in the treatment of anticlostridium. They verified that *B. intestinihominis* can indeed slow the growth of *C. difficile* through *in vitro* experimental validation (Steinway et al., 2015).

Theoretically speaking, the number of completed genome sequenced species should be as same as the number of corresponding GMNs. However, the current number of GMNs is much less than the number of sequenced species. The main reason is that the network construction pipeline still needs manual proofreading procedures due to the imperfect genetic annotation algorithm. In addition, the incomplete understanding of biochemical mechanisms also affects the development of metabolic networks (Wang et al., 2010).

### Genome-Scale Gene Transcriptional Regulatory Network

Gene transcriptional regulation is the most basic and important regulation mechanism in organisms. Therefore, computational analysis of the gene transcriptional regulation is helpful for the understanding of the interactions between transcriptional processes and TRNs, and could provide support for the understanding of the mechanisms of biological activities (De-nan, 2014).

The basic components of TRNs are the interactions between transcription factors (TFs) and the related target promoters which function in the activation or repression of gene transcription. In this definition, the intracellular signals that regulate TF activities or any other additional mechanisms that may influence the expression of genes were excluded, as well as the upstream environmental. Although the development of TRN is not as mature as that of GMN, the current TRN construction is more and more standardized and automated. The detailed construction method of the TRN in microorganism can be seen in this paper (Feist et al., 2009). The network construction method is roughly divided into four steps: Step 1: an automated genome-based construction with automated procedures and applying automated tools, such as SMILEY algorithm, GapFind/GapFill, and PathoLogic; Step 2: construction of the TRN based on bibliomic data or high-throughput data; Step 3: transforming a genome-scale reconstruction of the interactions into a computational model; Step 4: curation the network by adding physiological or *in vivo* experimental information to the genes and the network.

Transcriptional regulatory network is a very complex non-linear system. Therefore, it is difficult to be described in a mathematical model. So far, the studies of the TRN are still in the exploration stage in many aspects, and scientists are constantly exploring new and better ways to construct a more complete TRN. Using *Bacillus* as an example, in Sierro et al. (2008) improved the database of transcriptional regulation in *B. subtilis* (DBTBS), which is constructed in 1999 for collecting the information of experimentally characterized TFs, and they nearly doubled the information in DBTBS. Freyre-Gonzalez et al. (2013) examined each regulatory element that constituted the TRN of *B. subtilis* and presented some lessons from the construction processes. Arrieta-Ortiz et al. (2015) used the TRN of *B. subtilis* to calculate the activity of TFs with a new combination of composition analysis based on a large number of known transcriptome data and experimental data of *B. subtilis*. They predicted 2258 new regulatory interactions and recalled 74% previously known interactions with this model. The accuracy of predicted new regulation edges was 62% (391/635) (Arrieta-Ortiz et al., 2015). Faria et al. (2016) expanded a TRN for the central metabolism of *B. subtilis* reconstructed in 2008 by integrating the regulation information in DBTBS. They demonstrated that atomic regulons (ARs), which are the sets of genes with the same expression profile, are the effective references for improving the regulatory networks by finding the closely correlated genes in the ARs. The expanded model contains the regulatory information for 2500 of the 4200 genes in *B. subtilis* 168 (Faria et al., 2016). In addition, Gui et al. (2012) searched for the homologous TFs and their regulatory genes in the genetically closest pattern bacteria – *B. subtilis*, and used comparative proteomics to forecast a regulatory networks of *Bacillus pumilus*, which contains 195 TFs and 1201 controlled genes. The results of their study showed that comparative genomics is a reliable method to speculate the gene regulation network of some species based on the gene transcriptional regulatory relationships of their genetically close organism, which is the best and a widely studied model organism. This method offers a feasible way to explore some organisms’ regulatory networks without large-scale gene expression data (Gui et al., 2012).

The TRN can also be used to treat the human disease. Recently, Fowler and Galan (2018) built a regulatory network of *Salmonella typhi*, a pathogen causing typhoid fever. Typhoid fever, which is a frequently happened disease in human, was mainly caused by the typhus toxin secreted by *S. typhi*. Typhoid fever toxin is expressed uniquely by intracellular bacteria with unknown regulatory network. Fowler and Galan (2018) built the TRN of *S. typhi* and developed an algorithm called FAST-INSeq to identify the genes and mutants which influence the expression of typhoid toxins. This network can help to understand the expressional regulation of typhoid fever toxin in *S. typhi*, which would contribute to the treatment of typhoid fever (Fowler and Galan, 2018).

### Genome-Scale Signal Transduction Network

Signal transduction is an important cellular activity, a living cell can recognize, connect and interact with each other through signal transduction pathway, and realize the overall functional coordination and unity. Signal transduction carries plenty of biological functions, and is closely connected with the development of many diseases (Liu et al., 2008). In the early years, scientists believed that the STN is a linear cascade of information transmission and amplification. However, due to more studies of the system, scientists found that the concept mentioned above is incorrect. Therefore, a new view taking a STN as a system consisting of multiple complicated elements interacting in a multifarious fashion emerges. This view conflicts with the protein-centric or single-gene approach commonly used in the traditional research (Levchenko, 2003). Scientists found that except a few STNs that contain fewer signals and simpler network structures, such as Jak-STAT pathway, most STNs are fairly complex (Papin and Palsson, 2004). In the cellular signaling system, a large amount of phosphorylation and dephosphorylation reactions makes the signal transduction process usually reversible. The lacking of mass flow and the complexity of network state changes make the STN different from the GMN and TRN.

To determine the relationships between the mechanism and molecular regulations in STNs, it requires a large number of experiments. However, the standard single cell technique contributes little to the STN because the states of signal change dynamically and are different between individual cells (Kamps and Dehmelt, 2017). Fortunately, computational approaches such as bioinformatics analysis using known data and biological knowledge can help to interpret the STN (Shlomi et al., 2006). As early as Gomez et al. (2001) used a statistic model to calculate the molecular interactions in *Saccharomyces cerevisiae* on the basis of protein structure domain and network topology. This method can generate potential signaling pathways and also be applied to multiple species (Gomez et al., 2001). Rother et al. (2013) summarized the approaches of constructing a STN and classified them into three types: network topology-based method where network simulation could be applied using Boolean models, network specific-state based method where the network is simulated using differential equation models, and reaction-contingency based method where the network is simulated using agent based models, site-specific logical models or bipartite Boolean models (Rother et al., 2013). Each of the three methods performs well in small network modules. However, when the scale of network extended to the genome level, none of them is perfect for dealing with the whole information in the entire STN (Le Novere et al., 2009). In recent years, lots of small-scale STNs has been studied, such as the STN of HRas (Herrero et al., 2017), mTOC1 (Hoxhaj et al., 2017), cell circle (Wang et al., 2018), and cellular adhesion (Zheng et al., 2014). At the meantime, much more efforts are being made to construct large-scale STNs. Therefore, it is challenging to model the large STNs. Even though signaling network in bacteria is not as complex as those in eukaryotes, the construction of a large-scale STN is still a major challenge. Vinayagam et al. (2011) constructed a protein–protein interaction network to resembling the signal transduction flow between 1126 proteins, in which the interactions were obtained from yeast two-hybrid experiments of more than 450 signaling proteins. This network has been used to predict 18 previously unknown modulators in EGF/ERK signaling. Their results shows that the integration of genetic experiments and the computational approach is valuable for elucidating interactions between signaling proteins and facilities the identification of proteins in STNs (Vinayagam et al., 2011). Wang et al. (2011) also performed an approach called CASCADE_SCAN to construct STN with high-throughput data, which further showed that the high-throughput experiments are becoming a powerful tool for assisting in reconstructing large-scale STNs. Besides, the integration of different techniques such as optogenetics, protein design, surface patterning, and chemical tools was reported to provide some valuable information of the dynamic state of signals in the network and contribute in the construction of large-scale STNs (Kamps and Dehmelt, 2017).

## Integrated Networks in Microorgnisms

The establishment of various biological networks simulates and validates key activities in cells. With the recent advances in high-throughput studies, it has been realized that it is necessary to integrate different levels of biological information processing networks to fully investigate the biological mechanisms of the organisms (Kitano, 2002; Ryll et al., 2014). Therefore, the integrated network based on different network types has become a trend in the field of system biology and bioinformatics.

### Integrated Metabolic-Regulatory Networks

Metabolism and transcriptional regulation are two closely related cellular activities. Metabolites (substrates or reaction products) involved in metabolic reactions affect the activities of certain TFs or signal transduction pathways. On the other hand, enzyme-catalytic metabolic reactions are regulated by other genes or proteins, and the expression of enzymes is different in different environmental conditions. In recent decades, the integrative modeling of metabolic-regulatory networks has become an important research area in the modeling of microorganisms (Imam et al., 2015).

Covert et al. (2004) reconstructed the first genome-scale metabolic-regulatory integrated network of *E. coli* (*i*MC1010) based on the information derived from literature and databases. The network contains 906 metabolic genes and 104 regulatory genes, which regulate the expression of about 53% genes (479/906) in the *E. coli* metabolic network. This model is capable of predicting the previously unknown TFs, which play important roles in regulating metabolic processes, and interactions between metabolites and TFs (Covert et al., 2004). In 2005, they further used the literature-curated network *i*MC1010v1 to evaluate the performance of the functional states calculated in 15,580 growth environments for *E coli*. The results showed that the TRN responds mainly to the electron acceptors, which agrees with known experimental data. They also found that a complicated network had a small amount of dominant modes and the network clusters of activity profiles can be organized based on the activities of a few TFs. The integrated network gives crisper references than the single metabolic network for the further experiments to determine the functional states of an organism (Barrett et al., 2005).

Goelzer et al. (2008) reported a manually curated metabolic-regulatory integrated network of *B. subtilis*. The network includes post-translational regulations translational regulation, and modulation of enzymatic activities in the central metabolism. They decomposed the complex network into different locally regulated modules and found that these modules were managed by global regulators. Their results exhibited the functional organization of the metabolic-regulatory integrated network of *B. subtilis* (Goelzer et al., 2008).

Chandrasekaran and Price (2010) proposed an algorithm named probabilistic regulation of metabolism (PROM) and constructed a genome-scale regulatory-metabolic integrated network model for *E. coli* and *Mycobacterium tuberculosis*. Before this effort, another method named regulatory flux balance analysis (rFBA) has been used to integrate transcriptional regulatory with metabolic networks. rFBA used the Boolean logic to link transcriptional control to the metabolic process, which permits only on/off states of the network components (Shlomi et al., 2007). PROM introduces probabilities instead of Boolean rules to represent gene expression and the interactions between gene and TF (Simeonidis et al., 2013). The analysis of integrated *E. coli* network demonstrates that metabolic-regulatory integrated network is more accurate and comprehensive than the models constructed based on manual curation of literature. The integrated *M. tuberculosis* model incorporated data from more than 2,000 TF, 1,300 microarrays, 1,905 KO phenotypes and 3,300 metabolic reactions. The application of PROM on this model shows the capability of PROM on various organisms. Particularly, they demonstrated the outstanding capability of PROM in predicting the cellular phenotypes, drug targets, and functions of less studied regulatory genes.

Jiang et al. (2012) constructed a metabolic-transcriptory integrated network of *Corynebacterium glutamicum* by combining public databases and literature databases. The network contains 1,384 reactions, 1276 metabolites, 88 regulators, and 999 transcriptional regulations. The study systematically reorganized and analyzed the transcriptional regulation information of *C. glutamicum*, and expanded it to the metabolic network. They also preliminarily analyzed the metabolic network of *C. glutamicum* on the basis of the bow-tie structure of the network (Ma and Zeng, 2003). This work showed that the integration of the TRN and the metabolic network with the gene-enzyme-reaction relationship could be the foundation for the large-scale data integration and simulation analysis. The advantages of this integrated network are the discoveries of the relationships between transcription and metabolism in cells, which can’t be achieved if using either metabolic network or TRN only (Jiang et al., 2012).

Wang Z. et al. (2017) performed another algorithm called Integrated Deduced And Metabolism (IDREAM) to construct enhanced metabolic-regulatory integrated networks. IDREAM integrated Environment and Gene Regulatory Influence Network (EGRIN) models with the PROM framework. IDREAM performs better than PROM in the prediction of the phenotype and genetic interactions between TFs and metabolic processes in *S. cerevisiae* (Wang Z. et al., 2017).

Currently, large-scale metabolic-regulatory integrated network has been constructed for several microorganisms such as *E. coli* (Chandrasekaran and Price, 2010), *S. cerevisiae* (Herrgard et al., 2006), *Helicobacter pylori* (Schilling et al., 2002), *Phaeodactylum tricornutum* (Levering et al., 2017), comma shaped gram negative anaerobic bacteria (Mahadevan et al., 2006) and *C. glutamicum* (Kromer et al., 2004). Integration of metabolism and transcription processes is generally quite straightforward. Metabolic network produces precursors to synthesize the metabolites such as nucleotides and amino acids which are required by transcription processes. On the other hand, the TRN couples back to the metabolic network by managing the expression of the enzymes in the metabolic network and thus regulating the flux distribution among different metabolic functions (Feist et al., 2009).

### Integrated Regulatory-Signaling Networks

The integration of microbial transcriptional regulatory and signaling network is still in the preliminary stage. Wang and Chen (2010) combined the transcriptional regulation and signal transduction pathway (e.g., mainly presented in the form of protein–protein interaction) to construct the integrated yeast cellular network. The network connects these two networks together to form an integrated network using the nodes (i.e., TFs) between the TRNs and signaling pathways. The integrated cellular networks related to heat shock, hyperosmotic stress, and oxidative stress were constructed and the connections between these networks were further analyzed. With the hyperosmotic stress related network, the highly connected hubs related to the stress response were predicted. The analyses of these networks have identified a few TFs to serve as the core in the bow-tie structure and the essential elements for the rapid response to stress. In addition, they also identified a couple of genes/proteins related to stress responses or potential drug targets. This method, however, only integrates the transcriptional regulatory data with the protein–protein interaction in the signal transduction pathways, but not the completed STN. In order to get a more complete integration, it also needs to list all the components in a STN, and then combined with the TRN for the integration (Wang and Chen, 2010). Recently, Ignatius Pang et al. (2018) construct another regulatory-signaling integrated network of *S. cerevisiae* with protein–protein interaction as the bridge to link the regulatory (TF-gene pairs) and signaling (kinase-substrate pairs) parts. This network was used to investigate the negative genetic interactions and the genes in the negative genetic interactions closely related to the toxicity (Ignatius Pang et al., 2018).

In the study of algorithms, Roy et al. (2013) proposed a method called MERLIN (Modular regulatory network learning with per gene information) to reconstruct the regulatory network by identifying the connections from regulators, including proteins and TFs, to target genes. The regulatory network constructed by MERLIN actually reflects the integration of transcriptional regulation and signaling networks. The application of MERLIN on *S. cerevisiae* captured the co-regulatory relationships between downstream TFs and signaling proteins, and therefore uncovering the upstream signaling systems which control transcriptional responses (Roy et al., 2013). With the investigation of the integrated network, the regulation program of each gene in the human cells is much clearer than the application of either individual TRN or STN.

### Integrated Metabolic-Signaling Networks

The development of integrated network for metabolic and signaling networks is still in the very beginning stage. Few metabolic-signaling integrated networks have been published. Imam et al. (2015) discussed the challenges in the integration of these two network types. Firstly, signaling mechanisms are closely related to the specific concentrations of related molecules, while constraint-based approaches widely used in metabolic network analysis cannot reflect the metabolite concentrations. Secondly, lots of kinetic parameters are required in the construction of dynamic quantitative signaling network, but these parameters are rarely available. This aspect limits the integration of metabolic and signal transduction. Boolean or stoichiometric methods which do not require kinetics parameters or metabolite concentrations might be a possible choice for the integration of metabolic and signaling networks in the future.

### Integrated Metabolic-Regulatory-Signaling Networks

**FIGURE 1:**
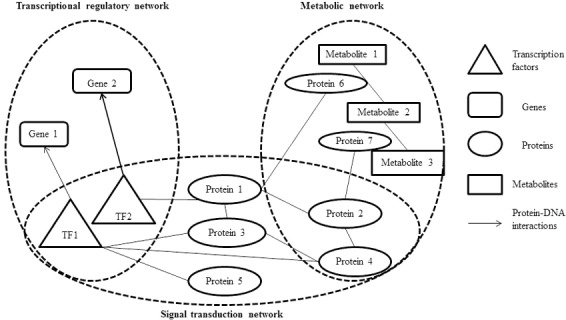
Graphic view of the integrated cellular network.

**FIGURE 2:**
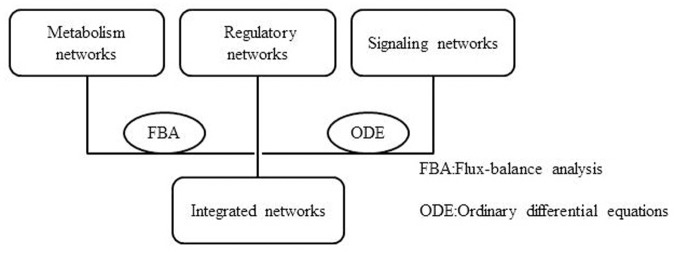
Schematic diagram of iFBA.

The integration of metabolic-regulatory-signaling networks is a challenge issue in the study of integrated networks. On the graphic view, there are common components (proteins or TFs) in metabolic, regulatory, and signaling networks (**Figure 1**). Therefore, it is theoretically possible to merge these three types of cellular networks into one integrated network. While actually, lots of elements should be considered in the integration process, such as the logics and computability. On a small scale network integration, Covert and Palsson (2002) developed a method named integrated FBA (iFBA) to model the dynamic behavior among metabolic, signaling, and regulatory networks. This method combines FBA with ordinary differential equations (ODE) and regulatory Boolean logic (**Figure 2**). They used this approach to construct an integrated network model of *E. coli* which combines a FBA based central carbon metabolic-regulatory network with an ODE based model of carbohydrate-uptaking-controlling network. They compared the prediction of *E. coli* single gene perturbation disturbance phenotypes and wild-type for diauxic growth on glucose/glucose-6-phosphate and glucose/lactose using rFBA and ODE methods. They found that iFBA is capable of identifying the dynamics of three transporters and three internal metabolites which cannot be predicted by rFBA alone. Furthermore, iFBA obtained different and more accurate phenotype predictions in the wild-type simulations and single gene perturbation simulations than the ODE model, which indicates that iFBA is an improvement over either individual rFBA or ODE method in network integration (Covert et al., 2008).

**FIGURE 3:**
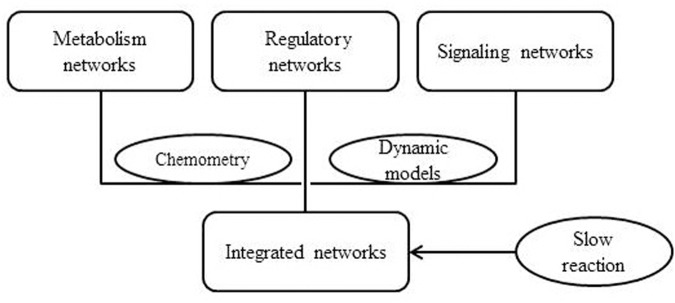
Schematic diagram of idFBA.

Lee et al. (2008) proposed a method called integrated dynamic FBA (idFBA) which could dynamically simulate cellular phenotypes with integrated networks. idFBA was applicable for the analysis of the integrated stoichiometric network of metabolic, regulatory, and signal transduction processes. In this method, the quasi-steady-state conditions were assumed for “fast” reactions and then the “slow” reactions was incorporated into the stoichiometric equation (**Figure 3**). idFBA has been applied to a representative small network of *S. cerevisiae*, in which metabolic, regulatory, and signaling activities have been included. Finally, idFBA got similar results with an equivalent kinetic model in the prediction of the influence of the extracellular environment on the cellular phenotypes. The advantage of idFBA is that it is capable of solving a linear programming problem without the detailed kinetic parameters, which makes it a possible approach for the genome-scale integration of metabolic, regulatory, and STNs (Lee et al., 2008).

For a large-scale network integration, Karr et al. (2012) collected information from 900 data sources, including reviews, books and databases, and constructed a whole cell model of *Mycoplasma genitalium*. This model includes data on metabolism, signal transduction and transcriptional regulation, and offers deep understanding on many previously unknown cellular behaviors, such as the inverse relationship between the replication rates and durations of DNA replication initiation. Furthermore, experimental analysis based on the model predictions has certified several previously undetected biological functions and kinetic parameters (Karr et al., 2012). However, due to the particularity of the species itself (e.g., unclear medium component, too small genome, etc.), the experimental data is rare, so the model was built using lots of data from other species, which makes it not suitable for other species. The good news is that Carrera et al. (2014) proposed a widely applicable modeling methodology for integrated network reconstruction and reconstructed an *E. coli* metabolic-regulatory-signaling integrated network by combining high-throughput transcriptome and phenomic data. The methodology is composed of four different algorithms including Expression Balance Analysis (EBA), flux Variability Analysis (FVA), TRAnscription-based Metabolic flux Enrichment (TRAME) and FBA, which were sequentially used to calculate the gene expression caused by the genetic or environmental perturbations, the flux balance bounds modified by the predicted gene expression, the metabolism-transcription interactions, and the optimized objective function under the modified flux bounds. With this methodology, the metabolism, transcription, and signal transduction information were integrated into one computable model. The application of this methodology on *E. coli* showed that the integrated network has a more powerful capability in phenotype prediction than the approaches using metabolic network alone (Carrera et al., 2014).

## The Integrated Networks of Microorganisms and Human Diseases

As many microorganisms are closely related to non-infectious human diseases, their biological networks naturally provide a possibility for studying the complex mechanisms of human diseases. For example, signal and metabolic network are usually used to understand the mechanism of disease and drug discovery (Hasan et al., 2012). In this point of view, another type of integrated network, microbe-disease association network integrated with microorganisms and human diseases, is also a quite helpful tool for improving the treatment of human diseases or development of new drugs. Up to date some efforts has been made to develop the algorithms or models for predicting the disease-related microorganisms based on the microbe-disease association network. Chen et al. (2017) developed a computational model KATZHMDA (KATZ measure for Human Microorganism–Disease Association prediction) based on an assumption that microorganisms with similar function likely to have similar interactions and non-interactions with diseases. With the similar assumption, Huang Y.A. et al. (2017) also developed a computational model called NGRHMDA (a neighbor- and graph-based combined recommendation model for human microbe-disease association prediction) to predict the association between microorganisms and diseases. They used a graph-based scoring method and neighbor-based collaborative filtering to calculate the possibility of association between microorganisms and diseases (Huang Y.A. et al., 2017). Huang Z.A. et al. (2017) developed a computational model PBHMDA (Path-Based Human Microorganism-Disease Association prediction) based on the Gaussian interaction profile kernel similarity calculation for microorganisms and diseases. Besides, this model also integrated the known microbe-disease relationships, and part of the results predicted with this model has been confirmed by previous published literature (Huang Z.A. et al., 2017). Similarly, Wang F. et al. (2017) proposed a semi-supervised computational model LRLSHMDA (Laplacian Regularized Least Squares for Human Microorganism-Disease Association) by integrating the Gaussian interaction profile kernel similarity and Laplacian regularized least squares classifier. This model got good performance on the prediction of chronic obstructive pulmonary, colorectal carcinoma, and asthma diseases in the case studies (Wang F. et al., 2017). No matter what kind of algorithms, the predictions were made based on the known knowledge of microorganisms and microbe-disease relationships. Therefore, as we know more about microbes and diseases, the computational models are expected to offer more insights in the identification of microbe-disease associations in the future.

## Future of Microbe Cellular Network

Construction and analysis of biological information processing-specific large-scale cellular networks (i.e., metabolic, signaling, and gene regulatory networks) has output many important biological insights in novel pathways, regulatory, and metabolic mechanisms. Given the fact that these networks are highly interconnected, the analysis of the integrated networks is expected to supply more novel understanding on biological behaviors which cannot be achievable using the biological information processing-specific network models alone. From biological information processing-specific networks to integrated network, it is an irresistible trend of the analysis of cellular networks. The integrated networks may provide better answers to the issues such as how transcription-regulatory interactions redirect flux distribution in a metabolic network; how a environmental or genetic disturbance influences the phenotype of an organism; or giving more accurate suggestions to the experiment designs and driving biotechnology applications. As lots of information is required in the reconstruction of a large-scale integrated networks, high-throughput experiments will play an increasingly significant role in the network integration. With the development of sequencing technology in recent years, many other types of cellular molecules involved in the regulatory process has been identified with high throughput experiment, and their related cellular networks have been studied, such as the network of mRNA, microRNA (Ferguson et al., 2018), lncRNAs (Zhang et al., 2018), and ceRNA (Xue et al., 2018). These small molecules participate in the regulatory network and control the RNA activity or gene expression directly or indirectly. Therefore, the integration of these molecules with TFs provides more information to the TRNs (Wong and Matus, 2017). With the involvement of more types of elements in the molecular networks, the integrated cellular networks will perform better to simulate the activity of the real cells. Although integrating of multiple types of information into a network will largely increase its complexity and calculation difficulties, the integrated network makes a computational network closer to a real cell, which pushes us go further from the dream of reproducing real creatures on computers.

## Ethics Statement

The study was approved by College of Life Sciences, Tianjin Normal Univeristy.

## Author Contributions

DW, LZ, and QW collected the references. EW and JS contributed in the guideline and revision of the manuscript. TH analyzed the reference. TH and DW wrote the paper.

## Conflict of Interest Statement

The authors declare that the research was conducted in the absence of any commercial or financial relationships that could be construed as a potential conflict of interest.
